# Nicotinamide riboside and pterostilbene reduces frequency and severity of undesirable symptoms of the menopause transition: an open-label, pilot clinical trial

**DOI:** 10.3389/fragi.2026.1773667

**Published:** 2026-05-13

**Authors:** Holly E. Holmes, Kamal Srivastava, Jennifer M. Scalici, Jyothi Dhuguru, Allyson E. Shea, Marie E. Migaud, Ryan W. Dellinger

**Affiliations:** 1 Elysium Health, New York, NY, United States; 2 Emory University School of Medicine, Atlanta, GA, United States; 3 Mitchell Cancer Institute, University of South Alabama, Mobile, AL, United States; 4 Department of Microbiology and Immunology, University of South Alabama, Mobile, AL, United States

**Keywords:** estradiol, estrone, hot flashes, menopause, NAD+ catabolome, NAD+ supplementation, nicotinamide riboside, pterostilbene

## Abstract

**Introduction:**

Nicotinamide adenine dinucleotide (NAD+) is a metabolite of vitamin B3 necessary for the production of key hormones like estradiol. Both NAD+ and estradiol levels decrease with age. Reduced estradiol levels have been associated with undesirable symptoms of the menopause transition. Previous clinical trials have demonstrated that oral supplementation with NRPT, a combination of the NAD+ precursor nicotinamide riboside (NR) plus pterostilbene (PT), significantly increases NAD+ levels. In the present study, the effects of a 7-day supplementation of NRPT on undesirable symptoms of the menopause transition were examined.

**Methods:**

An open-label, pilot clinical trial (ClinicalTrials.gov identifier NCT04841499) was conducted to assess the efficacy of NRPT supplementation (commercially known as Basis; a combination of nicotinamide riboside and pterostilbene) in 40 healthy women over 35 years of age, 32 of which self-reported symptoms associated with the menopause transition (MS group) and eight women who were not experiencing any (or minimal) symptoms associated with menopause (No-MS group). All 40 women were given the recommended dose of NRPT (250 mg NR and 50  mg PT) daily for 7 days. A menopausal symptom survey was taken at baseline and at the end of the 7-day intervention. Urine was also collected at baseline and after 7 days to assess levels of estrone (E1) and estradiol (E2) and the changes in the vitamin B3 catabolome stemming from NRPT supplementation.

**Results:**

At the end of the study, significant decreases in both the frequency and magnitude of bloating, hot flashes, and poor sleep were reported in the MS group as compared to baseline. This was accompanied by a significant increase in the E2/E1 ratio. The No-MS group did not report any significant changes in these endpoints. As anticipated NRPT intake significantly increased urinary levels of methyl-nicotinamide (Me-NAM) as well as the methylated pyridone carboxamides (Me-PYs) in all groups. A significant time x menopausal status interaction was observed for nicotinuric acid (NUA). No other significant changes occurred for the other nicotinamide catabolites, such as nicotinamide N-oxide (NAM-N-Oxide), and a newly characterized NAD^+^ catabolite, N-methyl-nicotinuric acid (Me-NUA).

**Discussion:**

This study demonstrates that NRPT is effective in significantly decreasing the frequency and magnitude of undesirable symptoms of the menopause transition and significantly increases the E2/E1 ratio in menopausal women.

## 1 Introduction

Menopause signals the end of the natural reproductive potential. Menopause typically occurs between the ages of 45 and 56 (Crandall et al., 2023). The time leading up to menopause, defined as 12 months past a woman’s last menses, is called perimenopause or the menopause transition. The menopausal transition is characterized by a diminishing pool of ovarian follicles accompanied by fluctuations in reproductive hormones (Santoro et al., 2021). In females of reproductive age, estradiol (E2) is the major circulating estrogen. E2 is essential to skeletal, vascular and energy homeostasis in women. Notably, the absolute concentration and relative levels of estrogens are affected during the menopause transition, with E2 levels plummeting. This results in estrone (E1) becoming the major circulating estrogen (Mauvais-Jarvis and Lindsey, 2024). E1 is considered a weak estrogen but is also the main source of E2 after menopause (Mauvais-Jarvis and Lindsey, 2024). The deficiency of E2 in post-menopausal women can lead to osteoporosis (Palacios et al., 2024), increased risk of cardiovascular disease (Anagnostis et al., 2022) and increased risk of type 2 diabetes (Paschou et al., 2024).

Menopause affects women at different stages of their adult life differently. However, three physiological responses to the menopausal transition are common to most women: bloating, hot flashes and poor sleep. Hot flashes occur in up to 80% of women and can last for 10 years after symptom onset (Gallicchio et al., 2015). Hot flashes are associated with discomfort, poor sleep, fatigue and a decreased quality of life (Ayers and Hunter, 2013). Hot flashes have also been linked to increased risks for cardiovascular disease (Thurston et al., 2008) and depression (Seritan et al., 2010). Furthermore, among women undergoing the menopause transition, those with relatively lower E2 levels experienced significantly increased frequency and severity of hot flashes (Gallicchio et al., 2015) and were associated with difficulties staying asleep (Haufe et al., 2022).

While hormone replacement therapy is used to compensate for the change in estradiol levels, the variable efficacy and associated risks with supplementation of exogenous estradiol are always a concern (Anagnostis et al., 2022; Manson et al., 2013; Paschou et al., 2024). Identifying novel treatments to address the undesirable symptoms and health risks associated with menopause transition-induced loss of E2 levels is of high clinical interest.

NRPT (commercially known as Basis) is a dietary supplement that contains the nicotinamide adenine dinucleotide (NAD+) precursor nicotinamide riboside (NR) and the antioxidant stilbenoid pterostilbene (PT). These two molecules have been shown to work synergistically in preclinical models (Isak et al., 2024; Obrador et al., 2021). NRPT has been shown to be safe in humans (Dellinger et al., 2023; Dellinger et al., 2017). In a double-blind, placebo-controlled, randomized trial among 120 generally healthy adults, NRPT taken daily at the recommended dose (250 mg NR and 50 mg PT) increased NAD+ levels in blood by 40% (Dellinger et al., 2017). The placebo group did not show changes in NAD+ levels throughout the study.

We hypothesize that potential alleviation of menopausal symptoms could be via the modulation of cofactors necessary for the reduction of E1 to E2. The reduced form of nicotinamide adenine dinucleotide monophosphate (NADPH) is the redox cofactor for the dehydrogenases responsible for the biosynthesis of most steroid-derived hormones, including E1 and E2. More specifically, 17-β hydroxysteroid dehydrogenase (17β-HSD) catalyzes the conversion of E1 to E2 using NADPH (Jin and Lin, 1999; Marchais-Oberwinkler et al., 2011). Levels of NADPH and its molecular precursor NAD+ decrease with age (Bradshaw, 2019; Migaud et al., 2024). Furthermore, systemic oxidative stress increases following menopause (Bourgonje et al., 2020) and endogenous antioxidant mechanisms require NADPH to combat the oxidative stress (Preethi et al., 2022); lowering the amount available for E1 conversion to E2. Thus, we propose that increasing NADPH levels in women undergoing the menopause transition would lead to increased endogenous E2 production. Circulating NAD+, and subsequently NADPH, levels can be increased by consumption of NAD+ precursors such as nicotinamide riboside (NR) (Migaud et al., 2024). In addition, endogenous cellular antioxidant responses can be bolstered and free radicals scavenged by PT (Acharya and Ghaskadbi, 2013; Nagarajan et al., 2022), which may protect NADPH levels. Furthermore, PT is naturally found in extracts of the *Pterocarpus soyauxii* heartwood tree. This extract has been shown to exhibit estrogenic and antioxidant activities in the prevention of postmenopausal symptoms in a surgically induced menopause rat model by providing vaginal stratification, improving lipid profile and insulin sensitivity (Mengue Ngadena et al., 2021), preventing neuropsychiatric disorders associated with menopause (Owona et al., 2024), and protecting against menopause-related nonalcoholic fatty liver disease (Owona et al., 2025).

In the current study, we investigated the efficacy of NRPT in 40 healthy women (32 of whom self-reported menopausal symptoms) in a 1-week open-label, pilot clinical trial. The primary objective of this study was to determine if NRPT supplementation can mitigate undesirable symptoms associated with the menopause transition. Prespecified secondary endpoints included the effects of NRPT on the natural production of E2, and assessments of safety and tolerability. As another secondary objective, we sought to determine whether the menopausal status was reflected in the urinary NAD^+^ catabolome and whether NRPT supplementation led to significant changes in its profile using liquid chromatography combined with mass spectrometry. In the process, we identified and characterized a yet unknown nicotinic acid catabolite, N-methyl-nicotinuric acid (Me-NUA).

## 2 Materials and methods

### 2.1 Clinical trial

This open label, pilot human clinical trial (ClinicalTrials.gov identifier NCT04841499) was carried out at the University of South Alabama and conducted in accordance with the protocol and with the consensus ethical principles derived from international guidelines including the Declaration of Helsinki and Council for International Organizations of Medical Sciences International Ethical Guidelines. Applicable International Council for Harmonization (ICH) Good Clinical Practice (GCP) Guidelines were also followed. The study protocol was approved by the University of South Alabama Institutional Review Board (Mobile, AL) on 23 February 2021 (IRB number: 1520213-6/19-454)**.** Recruitment, onsite monitoring, data collection and data management were provided by the Mitchell Cancer Center (Mobile, AL). The study recruited participants from April 2021, and the in-human phase of the trial was completed by July 2021.

### 2.2 Participants

Generally healthy females aged 35 years and over were recruited from the faculty and staff pools at the Mitchell Cancer Institute, the Children’s and Women’s Hospital, and the Strada Health Center. As the aim of the study was to examine the effect of NRPT on undesirable symptoms associated with the menopause transition, the majority of women who were enrolled self-reported menopausal symptoms (MS group). A smaller group of women not experiencing menopausal symptoms (No-MS group) were also enrolled to act as a control cohort. The target ratio of individuals enrolled into the MS group vs. the No-MS group was 3:1. A total of 40 women were recruited: 32 women in the MS group and eight women in the No-MS group. A similar paradigm was used preciously to examine the effect of exogenous estradiol on poor sleep and hot flashes in women undergoing the menopause transition (Joffe et al., 2011). Participants were excluded if they were undergoing hormone replacement therapy, taking supplements for treatment of symptoms of the menopause transition, pregnant or lactating, active in another clinical trial, undergoing any active cancer treatments, or using immunosuppressive drugs. Participants were instructed not to take any Vitamin B3 supplements for 7 days prior to study initiation or during the study. All participants provided written informed consent to participate in the study.

### 2.3 Intervention

The investigational product (IP) NRPT (commercially known as Basis) contained 125 mg of NR and 25 mg PT per capsule. All 40 participants were provided with the IP at the baseline clinic visit (Visit 2) and were instructed to take two capsules (total dose of 250 mg NR and 50 mg PT) orally per day for 7 days. Each capsule contained the excipients microcrystalline cellulose, silicon dioxide and magnesium stearate. Participants were also instructed not to alter their daily routines.

### 2.4 Clinic visits

Participants attended three in-clinic visits: one screening visit (Visit 1; up to 4 weeks prior to the baseline visit), followed by a baseline (Visit 2; Day 0) and one end-of-study visit (Visit 3; Day 7) with subsequent telephone follow up 2 weeks after the last clinic visit. All clinic visits were done in the morning. During the baseline visit, participants completed a questionnaire regarding their menopausal symptoms, ranking the frequency and severity of their bloating, hot flashes and poor sleep on a scale of 0 (no symptoms) to 9 (symptomatic) (Supplementary Figure 1). Demographic data (age, race and ethnicity) was also collected at the baseline visit. A urine sample was also collected from all participants (in the morning). Starting from Visit 2, participants took their assigned study product once daily in the morning for 7 days. At the end of the study (Visit 3), all baseline procedures were performed again.

### 2.5 Outcomes

The primary outcome measures were the 7-day change in undesirable symptoms of the menopause transition, and the 7-day change in E2 production, measured in urinary waste.

### 2.6 Investigational product accountability and safety reporting

IP accountability was monitored by onsite visits. All participants were given a 7-day supply of the IP at Visit 2 and told to take 2 capsules each morning for 7-day. Safety information was gathered systematically by the investigator team according to the specifications contained within the study protocol.

### 2.7 Detection of E1 and E2

Estrone (E1) and estradiol (E2) concentrations in the urine were measured according to recommended protocols using the E1 immunoassay kit from Aviva Systems Biology (San Diego, CA) and the DetectX estradiol immunoassay kit from Arbor Assays (Ann Arbor, MI), respectively. The measurements were carried out in technical triplicates and normalized to urine creatinine levels measured by LC-MS.

### 2.8 Synthesis and characterization of methyl-nicotinuric acid

Methyl iodide (367 mg, 2.58 mmoles) was added to an anhydrous methanolic solution of nicotinuric acid (100 mg, 0.55 mmoles; 3 mL solution). The mixture was heated at reflux overnight. Methanol was then removed under reduced pressure and crude N-methyl nicotinuric acid (Me-NUA) was purified by crystallization using dichloromethane and methanol to afford the desired compound as yellow crystals in quantitative yield:^1^H NMR (400 MHz in CD_3_OD) ppm: δ 9.38 (s, 1H), 9.05 (d, J = 5.7 Hz, 1H), 8.94 (d, J = 8.2 Hz, 1H), 8.20 (t, J = 7.0 Hz, 1H), 4.48 (s, 3H), 4.11 (s, 2H); ^13^C NMR (100 MHz in CD_3_OD) ppm: δ 171.0, 161.8, 147.7, 145.9, 143.1, 133.4, 127.9, 48.7, 42.7. HRMS calculated for M^+^(C_9_H_11_N_2_O_3_^+^), m/z 195.0769, observed 195.0761.

### 2.9 LC-MS analyses of the urinary NAD catabolome

For each sample collection, 5 mL of urine were freeze-dried upon receipt and stored at −80 °C until sample processing, extraction, and analyses, at which time the extraction was performed by adding methanol (4 mL) and chloroform (2 mL) in a 15 mL falcon tube and vortexed for 5-10 s. Two milliliters of distilled water and 2 mL of chloroform were sequentially added. The resulting suspension was vortexed and then centrifuged. Six hundred microliters of polar phase were transferred to an Eppendorf tube and dried on a speed vac concentrator (without heat). The residue was dissolved into 50 µL H_2_O containing 10 µM labeled Me-NAM (^13^C^12^C_6_H_6_D_3_N_2_^18^O) and 1 µM labeled NAM (C_6_H_6_N_2_^18^O). The sample was vortexed well and centrifuged 13,000 g for 15 min 40 μL were transferred to the MS vial for analysis. The samples were analyzed on a Thermo Q Exactive Plus instrument combined with an Agilent LC system fitted with a Zorbax 300SB-C18 (2.1 × 150 mm) column and guard column to which was applied a gradient of Solvent A (10 mM ammonium acetate in H_2_O + 0.1% formic acid) and Solvent B (MeOH + 0.1% formic acid) with a total running time of 38 min. Data collection was performed under positive ionization only. Each sample was analyzed with a 1.0 µL injection volume. A blank was run between each sample to minimize and monitor for carry-over. A five-point standard curve ranging from 1.0 to 100 µM was obtained using a solution mix of external non-labeled standards. Spiked heavy labeled internal standards of NAM (1.0 µM) and Me-NAM (10.0 µM) were also used to estimate the µM concentration of the non-labeled compounds. Targeted SIM scans and PRM scans were performed to provide MS1 data for quantitation and MS2 data for validation. We quantified nicotinamide (NAM), N-methyl nicotinamide (Me-NAM), nicotinuric acid (NUA), nicotinamide N-oxide (NAM-N-oxide), N-methyl-pyridone carboxamides (Me-PY), pyridones carboxamides (PY), and pyridone carboxamide ribosides (PYR) in urine normalized to creatinine content. A novel nicotinic acid catabolite was identified as N-methyl-nicotinuric acid (Me-NUA) and quantified. Once normalized to the internal standard, each metabolite’s relative changes in AUC values have been used in subsequent statistical analyses.

### 2.10 Statistical methods

For the menopausal symptoms analyses, paired comparisons were performed using the Wilcoxon matched-pairs signed-rank test due to the ordinal nature of the data, and the results are presented as median and interquartile range (IQR). For the E2/E1 analyses, unpaired t-tests were used to identify intergroup differences, and paired t-tests were used to identify intragroup differences. For metabolomic analyses, data distributions were assessed using a Shapiro-Wilk test. Because several metabolites did not meet assumptions of normality, non-parametric paired tests (Wilcoxon matched-pairs signed-rank test) were initially used for within group comparisons. To evaluate the effects of time (baseline vs. 1 week) and menopausal status, data were reanalyzed using mixed effects models (REML), with time treated as a repeated factor and menopausal status as a between-subject factor. A two-way repeated-measures ANOVA was also performed and interpreted with caution due to deviations from normality and variability in the data. When a significant interaction was detected, *post hoc* pairwise comparisons were performed using the same model; otherwise, only main effects were reported. Statistical analyses were performed using Prism 10.3.1 (GraphPad Software, LLC). p < 0.05 was considered statistically significant for all analyses.

## 3 Results

### 3.1 Trial overview

**FIGURE 1:**
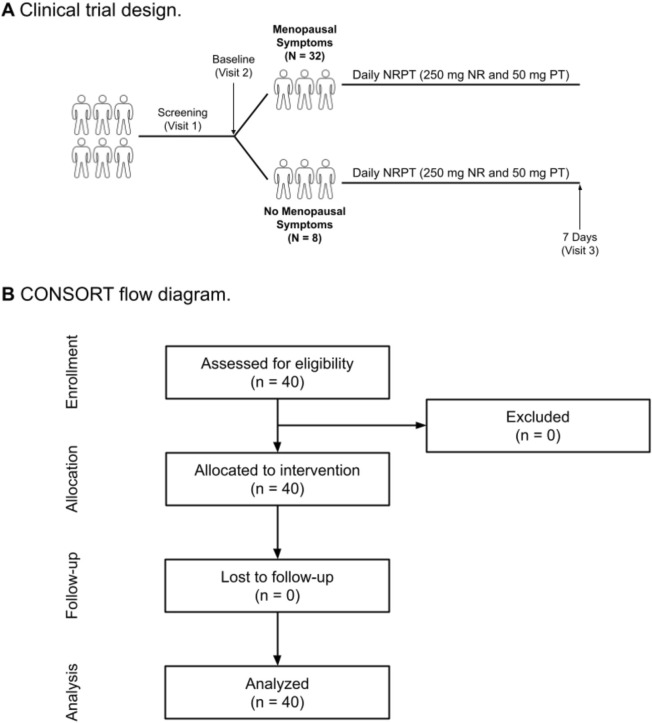
Clinical trial diagrams. **(A)** Clinical Trial Design diagram. Schematic depicting the open label two arm parallel group study. The study consisted of a 1-week intervention. Clinic visits occurred at screening, Day 0 (baseline) and Day 7 (end of study). **(B)** Clinical Trial Flow Design. Schematic depicting recruitment and disposition of study participants. A total of 40 potential participants were screened to enroll 40 eligible participants that were allocated to the intervention group. Forty participants completed the trial.

In this open-label, pilot clinical trial, the efficacy of NRPT as a treatment for the undesirable symptoms of the menopause transition was examined. 40 participants were screened and determined to be eligible for the study. All 40 were enrolled in the study. Of those 40 participants, 32 self-reported experiencing menopausal symptoms (MS group) and eight were experiencing no (or minimal) menopausal symptoms (No-MS group). The No-MS group served as a control group. All participants took NRPT for 7 days, with evaluations at baseline and at the 7-day timepoint. No participants were lost to follow-up. All 40 participants were analyzed for the primary endpoints. A schematic of the study and a CONSORT flow diagram are shown in Figure 1.

### 3.2 Baseline characteristics

The baseline characteristics for the participants enrolled in the study are presented in Table 1. The MS group was significantly younger than the No-MS group (48.3 years vs. 57.5 years, p < 0.01). The race and ethnicity characteristics of the two groups were similar. All 40 participants completed a questionnaire to evaluate the frequency and unpleasantness of menopausal symptoms (Supplementary Figure 1) at baseline. The MS group scored significantly higher on both the frequency and unpleasantness of bloating, hot flashes, and poor sleep than the No-MS group. Owing to the differences in the primary endpoints, we focused our investigation on intra-group analyses (rather than inter-group analyses, which would have been confounded by the baseline differences).

**TABLE 1:** Baseline characteristics of study participants. Age is given as ‘Mean (SD)’. An unpaired t-test was used to determine intergroup differences. Scored values are given as ‘Median (IQR)’and a Wilcoxon matched-pairs signed-rank test was used to determine intergroup differences.

Characteristic	Menopausal symptoms (n = 32)	No menopausal symptoms (n = 8)	P value
Age, years	48.3 (6.7)	57.5 (9.1)	0.0024
Race, n (%)	​	​	​
White	19 (59)	5 (63)	-
African American	11 (34)	2 (25)	-
Asian	1 (3)	1 (13)	-
Hispanic, n (%)	1 (3)	0 (0)	-
Vitamin use, n (%)	17 (53)	6 (75)	-
Bloating	​	​	​
Frequency	4.0 (3.0, 7.0)	1.0 (0.0, 3.5)	0.0246
Unpleasantness	4.5 (3.0, 7.25)	1.0 (0.0, 3.25)	0.0147
Hot flashes	​	​	​
Frequency	5.5 (4.0, 7.0)	0.0 (0.0, 1.25)	0.0003
Unpleasantness	5.0 (4.0, 7.25)	0.0 (0.0, 1.25)	0.0005
Poor sleep	​	​	​
Frequency	7.0 (5.0, 8.0)	5.0 (0.0, 5.25)	0.0141
Unpleasantness	7.0 (5.0, 8.0)	3.5 (0.0, 5.75)	0.0175

### 3.3 Bloating, hot flashes and poor sleep

**FIGURE 2:**
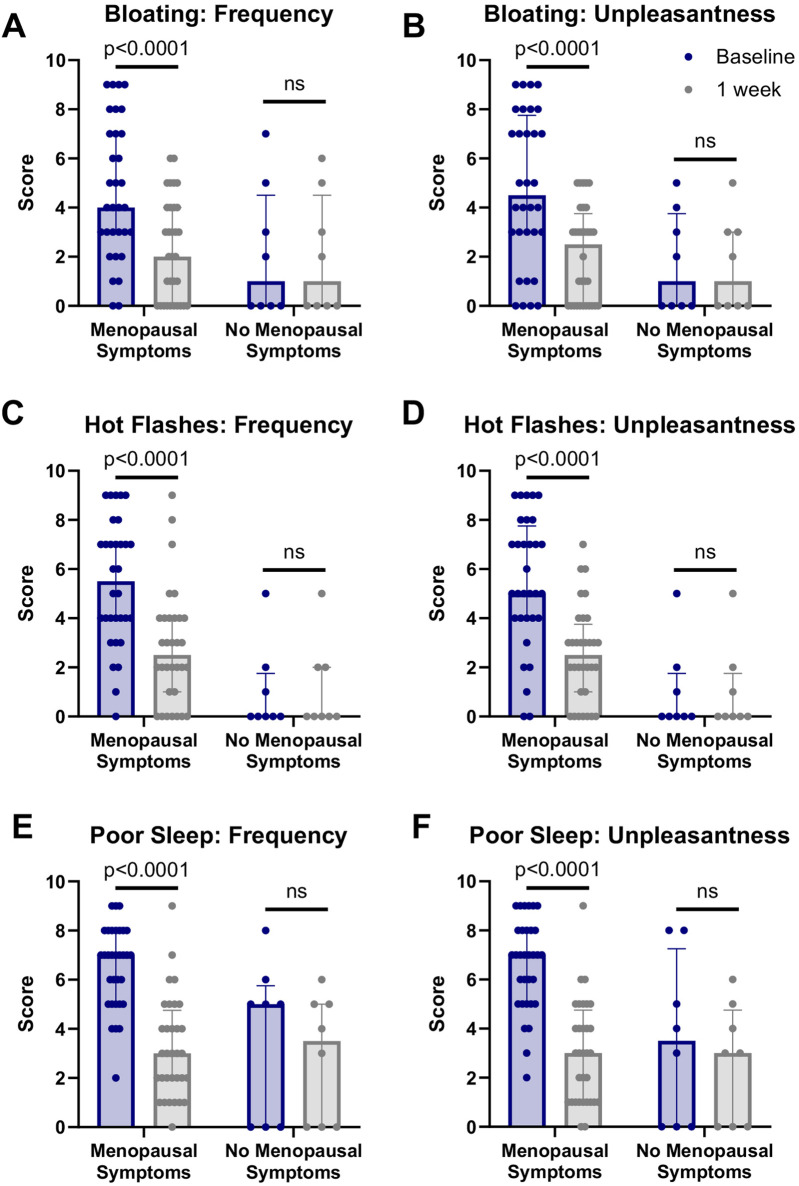
NRPT improved symptoms of menopause in MS group. Data from the menopausal symptom questionnaire is presented for **(A)** frequency and **(B)** unpleasantness of bloating, **(C)** frequency and **(D)** unpleasantness of hot flashes, and **(E)** frequency and **(F)** unpleasantness of poor sleep. Data are shown as individual values with median and interquartile range (IQR) for each group. Paired comparisons between baseline and 1 week were performed using the Wilcoxon matched-pairs signed-rank test. Significant improvements were observed in the menopausal symptoms group (p < 0.0001), whereas no significant changes were detected in the no menopausal symptoms group (ns).

The primary outcome measures included the 7-day change in undesirable symptoms of the menopause transition. In the MS group, significant decreases in both the frequency and unpleasantness of bloating, hot flashes, and poor sleep were observed at day 7 compared to baseline (p < 0.0001 relative to baseline for all comparisons) (Figure 2). Notably, the majority of the symptom scores at the 7-day timepoint were now within the ranges of the No-MS group, with only the unpleasantness of hot flashes remaining significantly different between the groups (Supplementary Table 1).

We also investigated the proportion of subjects in each group who reported an improvement in menopausal symptoms after 7 days of NRPT treatment. 78% of the MS group reported improvements in both frequency and unpleasantness of bloating (Table 2). For hot flashes, 75% of the MS group reported an improvement in the frequency of hot flashes while 84% of the MS group reported an improvement in the unpleasantness of their hot flashes. For sleep, 84% of the MS group reported an improvement in the frequency of poor sleep and 91% of the MS group reported an improvement in the unpleasantness of poor sleep (Table 2).

**TABLE 2:** Proportion of subjects who reported an improvement in menopausal symptoms. All values are given as ‘n (%)’.

Symptom	Menopausal symptoms (n = 32)	No menopausal symptoms (n = 8)
Bloating	​	​
Frequency	25 (78)	1 (13)
Unpleasantness	25 (78)	1 (13)
Hot flashes	​	​
Frequency	24 (75)	1 (13)
Unpleasantness	27 (84)	2 (25)
Poor sleep	​	​
Frequency	27 (84)	3 (38)
Unpleasantness	29 (91)	2 (25)

No significant differences were observed in any symptom at day 7 compared to baseline in the No-MS group (Figure 2), nor did a meaningful proportion of this group report symptom improvements (Table 2).

### 3.4 Estrone and estradiol

**FIGURE 3:**
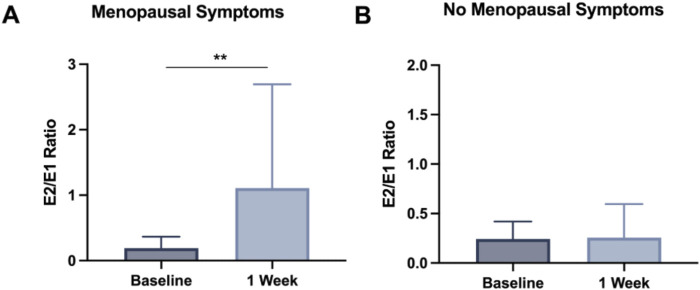
NRPT increased the E2/E1 ratio in the MS group. Urine was analyzed for estrone (E1) and estradiol (E2) concentrations at baseline and after 7-day of supplementation with NRPT. The E2/E1 ratio at baseline and after 7-day of NRPT supplementation is presented for **(A)** the MS group and **(B)** the No-MS group. Participants with E2/E1 ratios >2 were excluded as these are atypical of peri-menopausal and post-menopausal women (n = 4). The graphs show the mean and standard deviation for each group. A paired t-test was used to determine differences. **p < 0.01**.**

The primary outcome measures also included the 7-day change in E2 production, measured in urinary waste. Urine was collected from all study participants at baseline and after 7-day supplementation with NRPT. The concentrations of E1 and E2 were then analyzed in urine samples and the E2/E1 ratio was calculated for each participant. We first considered all available data and did not identify any significant differences in either the MS or the No-MS groups (Supplementary Figure 2). However, closer examination of the data revealed that 4 of the 40 participants had an E2/E1 ratio >2 at baseline, all of them in the MS group. This was unexpected, given that during the aging process E2 production is reduced and E1 becomes the major circulating estrogen (Mauvais-Jarvis and Lindsey, 2024). The four participants with E2/E1 ratios greater than 2 (indicating that E2 is still their major circulating estrogen) were excluded and the analysis was repeated. As shown in Figure 3, 1 week of supplementation with NRPT significantly increased the E2/E1 ratio in the MS group compared with baseline (p < 0.01), while the E2/E1 ratio was unaffected in the No-MS group. Furthermore, the 7-day change in E2 levels trended upward with respect to baseline (p = 0.05), while the 7-day change in the levels of E1 trended downward with respect to baseline (p = 0.09) in the MS group (Supplementary Figure 3). The results became statistically significant when looking at the E2/E1 ratio.

### 3.5 Vitamin use

All participants were asked about their vitamin use at the start of the study. Within the MS group, 17 women reported vitamin use and 15 women reported no vitamin use (Table 1). We next examined whether vitamin use was a determinant in response to NRPT, focusing on participants in the MS group who reported total alleviation of at least one symptom after 7 days of supplementation (i.e. a score of 0 at the end of the study). For bloating, over half of the MS group who reported vitamin use experienced a complete alleviation in the frequency (53% of subgroup) and unpleasantness (63% of subgroup) of their bloating (Table 3). In contrast, only 1 participant in the no vitamin use (7% of subgroup) reported complete alleviation of frequency of bloating. For hot flashes, over a third of the MS subgroup who reported vitamin use experienced a complete alleviation of both the frequency (35% of subgroup) and unpleasantness (35% of subgroup) of their hot flashes (Table 3). Only 1 participant who reported no vitamin use (7% of this MS subgroup) experienced complete alleviation in both the frequency and unpleasantness of their hot flashes. Vitamin use appeared to have less impact on poor sleep with only 6% of the MS subgroup reporting total improvement in the frequency of poor sleep, while 12% of the MS subgroup reported total improvement in unpleasantness of poor sleep (Table 3). No participant of the MS subgroup who reported no vitamin use experienced complete alleviation of either the frequency or severity of poor sleep.

**TABLE 3:** Proportion of subjects with menopausal symptoms who reported a complete alleviation of menopausal symptoms, categorized according to their reported vitamin use. All values are given as ‘n (%)’.

Symptom	Vitamin use (n = 17)	No vitamin use (n = 15)
Bloating		
Frequency	9 (53)	1 (7)
Unpleasantness	11 (65)	0 (0)
Hot flashes		
Frequency	6 (35)	1 (7)
Unpleasantness	6 (35)	1 (7)
Poor sleep		
Frequency	1 (6)	0 (0)
Unpleasantness	2 (12)	0 (0)

### 3.6 Adverse events

No adverse events were reported during this study.

### 3.7 The urinary NAD+ catabolome profile pre and post 7-day of NRPT

**FIGURE 4:**
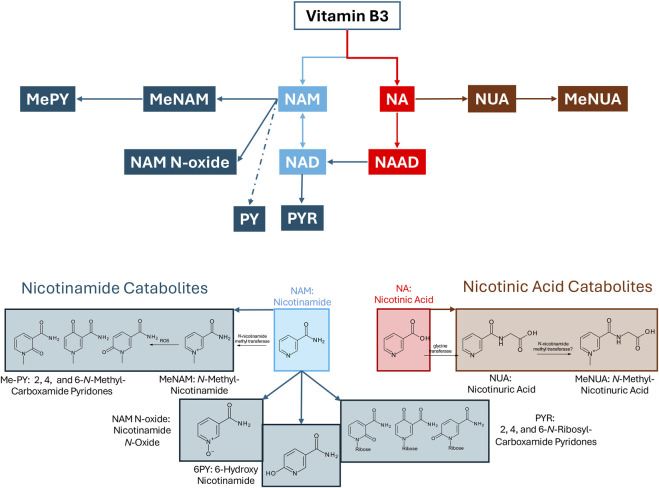
Schematic showing Vitamin B3 metabolites including the structures for the NAM and NA catabolites.

**FIGURE 5:**
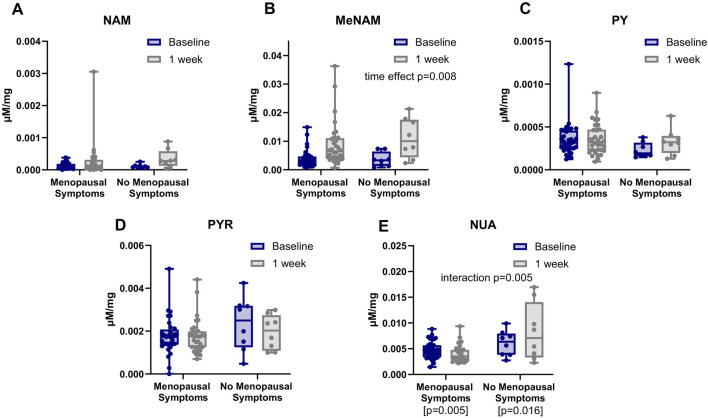
NRPT increases MeNAM and alters NUA in a menopause-dependent manner. Concentrations of NAD+ catabolites normalized to urinary creatinine at baseline and after 1 week of NRPT supplementation for **(A)** nicotinamide (NAM), **(B)** N-methyl-nicotinamide (MeNAM), **(C)** 6-hydroxynicotinamide (PY), **(D)** 2,4 and 6-N-ribosyl-3-carboxamide pyridone (PYR), and **(E)** nicotinuric acid (NUA). Data are stratified by menopausal symptom status. Statistical analysis was performed using a mixed-effects model (REML) with time (baseline vs. 1 week) as the repeated factor and menopausal status as the between-subject factor. A significant main effect of time was observed for MeNAM. A significant time x menopausal status interaction was observed for NUA. Post hoc pairwise comparisons showed that NUA changed from both baseline to 1 week in both groups, with a greater change in the participants with menopause symptoms. No significant effects were observed at baseline, whereas a difference emerged at 1 week. No significant effects were seen for NAM, PY or PYR.

The urinary NAD+ catabolome profile was measured for all participants at Day 0 (baseline) and following 1 week of NRPT administration (Day 7). A schematic depicting the metabolites of Vitamin B3 is shown in Figure 4. The chemical structures of nicotinamide (NAM) and nicotinic acid (NA) are also depicted along with their respective catabolites (Figure 4). Included in this schematic is the newly identified N-Methyl-Nicotinuric Acid (Me-NUA). Synthesis, NMR, and LC/MS characterization of Me-NUA is provided here in the Materials and Methods section as well as Supplementary Figures 4–8. The absolute quantities for NAM, N-methyl-nicotinamide (Me-NAM), nicotinuric acid (NUA), 6-hydroxynicotinamide (PY), and 2,4 and 6-N-ribosylcarboxamide pyridones (PYR) are stratified by subgroup (Figure 5). As expected, Me-NAM levels increased following NRPT supplementation (Figure 5), with a significant main effect of time observed independent of menopausal status. This is consistent with previous clinical reports and is indicative of taking a supplement containing NR (Elhassan et al., 2019; Trammell et al., 2016). In contrast, NUA responded differently to supplementation depending on menopausal status, with a greater increase observed in participants with menopausal symptoms. No other NAD+ catabolites shown in Figure 5 exhibited any significant changes.

**FIGURE 6:**
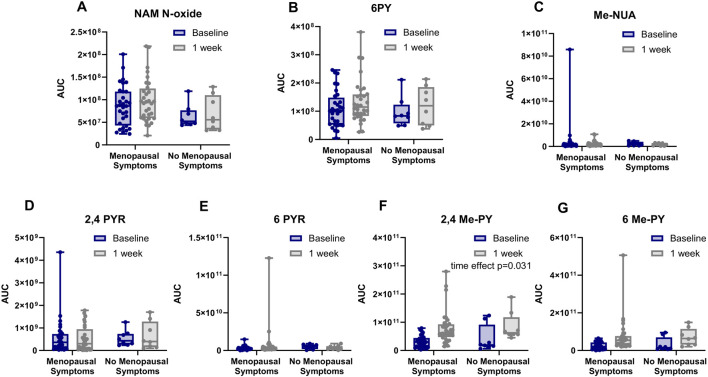
NRPT increased 2/4 Me-PY in all participants. ΔAUC values of NAD+ catabolites in urine at baseline and after 1 week of NRPT supplementation for **(A)** nicotinamide-N-oxide (NAM-N-oxide), **(B)** 6-hydroxynicotinamide (6PY), **(C)** N-methyl-nicotinuric acid (Me-NUA), **(D)** N-ribosyl-3-carboxamide 2/4-pyridone (2/4 PYR), **(E)** N-ribosyl-3-carboxamide 6-pyridone (6 PYR), **(F)** N-methyl-3-carboxamide 2/4-pyridone (2/4 Me-PY) and **(G)** N-methyl-3-carboxamide 6-pyridone (6 Me-PY). Data are stratified by menopausal status. Statistical analysis was performed using a mixed-effects model (REML) with time (baseline vs. 1 week) as the repeated factor and menopausal status as the between-subject factor. A significant main effect of time was observed for 2/4 Me-PY. No significant time x menopausal status interactions or menopause effects were detected for any other metabolite.

Upon refining the LC-MS analyses and acquiring additional external standards, we were able to differentiate the isomeric distribution between the 6-PYR and the 2/4-PYR ([M + H+], m/z @ 271) and differentiate 6-PY from nicotinamide-N-oxide (NAM-N-oxide) ([M + H+], m/z @ 139) as well as differentiate N-methyl-3-carboxamide 6-pyridones (6-Me-PY) form N-methyl-3-carboxamide 2/4-pyridone (2/4-Me-PY) ([M + H+], m/z @ 153). We were also able to detect and measure Me-NUA’s AUC values ([M + H+], m/z @ 195). Upon examination of the normalized AUC values of the wider pool of catabolites, 2/4-Me-PY increased following 7 days of NRPT supplementation (Figure 6). No other NAD + catabolites showed significant changes (Figure 6).

## 4 Discussion

**FIGURE 7:**
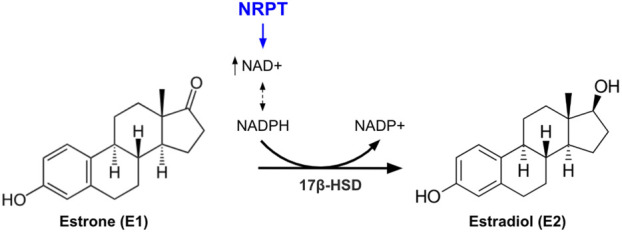
Schematic showing proposed mechanism of action of NRPT. Oral NRPT increases the availability of NAD+ and NADPH, which subsequently increases the activity of the NADPH-dependent enzyme 17β-hydroxysteroid dehydrogenase (17β-HSD). This leads to increased conversion of estrone (E1) to estradiol (E2).

In this study, we evaluated the impact of 7-day administration of NRPT on undesirable symptoms of the menopause transition. In the MS group, significant reductions in the frequency and unpleasantness of bloating, hot flashes and poor sleep were reported. These findings were accompanied by a significant increase in the E2/E1 ratio. Thus, we propose a mechanism by which supplementation with NRPT increases circulating NAD+ levels, which leads to a subsequent increase in NADPH levels, which in turn promotes 17β-HSD activity reducing E1 to E2 (Figure 7). This model is further supported by the observed increase in E2 levels following NRPT supplementation in the MS group coupled with a decrease of E1 levels in response to NRPT in the MS group.

Our findings of relief from unpleasant menopausal symptoms after just 7 days are surprising, but not unexpected. Previous clinical studies have demonstrated that supplementation with NR results in increased blood levels of NAD+ in as few as 8 days (Airhart et al., 2017). In addition, a single oral dose of NR at 100 mg, 300 mg or 1,000 mg demonstrated dose dependent increases in NAD+ metabolism in blood of healthy adults (Trammell et al., 2016). Although it is not known how quickly the increase in NAD+ availability translates to increased E2 production, our findings of symptomatic relief coupled with an increase in the E2/E1 ratio suggest the physiological effects are rapid.

One apparent discrepancy in our data is that while we observed a significant increase in the E2/E1 ratio in the MS group, we did not see a similar significant increase in the No-MS group. E1 was the predominant estrogen for all participants analyzed, so it is unclear why the E2/E1 ratio would not go up for all study participants. The answer may lie with the expression of the enzyme responsible for converting E1 to E2, 17β-HSD. The expression of 17β-HSD has been reported to decrease in post-menopausal women, reaching its lowest levels at about 10 years post menopause (Brodowska et al., 2014). Since the average age of the No-MS group was 9 years older than the MS group, our data are consistent with a decreased expression level of 17β-HSD in the No-MS group which could explain this lack of response to NRPT.

Supplementation with NRPT was also reflected in changes in the abundance of several NAD+ catabolites. As expected, Me-NAM increased following NRPT administration. In the broader AUC-based metabolite panel, 2/4-Me-PY also increased following NRPT supplementation. These increases are consistent with previous reports as indicative of supplementation with NR in all participants (Elhassan et al., 2019; Trammell et al., 2016). These data served as confirmation that the NRPT supplement was taken by each participant. In contrast, NUA showed a menopause-dependent response to supplementation, with a greater increase in participants with menopausal symptoms. No other significant effects were observed in the NAD+ catabolites analyzed.

Additionally, this NRPT supplementation investigation broadened the pool of NAD+ catabolites that can be characterized in human urine. NUA is not a catabolite commonly reported in the NAD+ literature since it is rarely observed in murine biology. However, NUA and nicotinamide N-oxide are NAD+ catabolites easily detected in human physiology. Furthermore, the detection and characterization of the methylated form of nicotinuric acid (Me-NUA) reveals that not only is NUA a substrate for a N-methyl transferase, maybe even N-nicotinamide methyl transferase (NNMT), but also a potential substrate for oxidation.

Another interesting finding of this study is the high percentage of participants that reported positive responses in the MS group and the magnitude of those positive effects. For example, 75% of the participants in the MS group reported a reduction of hot flash frequency and 21% of the MS group reported complete alleviation of hot flashes after 7-day supplementation with NRPT. In comparison, a recent clinical study looking at the use of ashwagandha root extract (ARE) supplementation for treatment of hot flashes during the menopause transition demonstrated a significant decrease in hot flash frequency of 27% after 56 days (Vani et al., 2025). Specifically, the number of hot flashes per day went from 14.5 to 10.6 with no data reported on if any of the 30 participants treated with ARE experienced complete alleviation of hot flashes (Vani et al., 2025). Since ARE is currently marketed as a supplement for relief of symptoms associated with the menopause transition, NRPT may provide another safe and effective choice for these women, especially for relief from hot flashes.

This study has several limitations that warrant discussion. Firstly, we focused our investigation on bloating, hot flashes, and poor sleep–yet there are additional menopausal symptoms such as brain fog, fatigue, and mood swings that remain unexplored. It would be prudent to employ a clinically validated menopausal symptom survey, such as the Menopause-Specific Quality of Life Questionnaire, in future studies. While the average age of the MS group (48.3 years old) was within the typical age range when the menopause transition occurs, we did not include detailed menstrual cycle information in our inclusion criteria for this pilot study. Thus, we included participants in perimenopause or menopause as long as they were symptomatic. Future studies should distinguish between different stages of the menopause transition. We also observed that participants who reported taking vitamins seemed to respond better to NRPT treatment than those who did not take vitamins, with a greater proportion of vitamin takers experiencing a complete alleviation of menopausal symptoms. However, without more detailed knowledge of the products each participant was using, it is impossible to determine whether there were specific active ingredients that could be causing the enhanced effects. Finally, we acknowledge that this is an open-label pilot study with a 1-week intervention. Future placebo-controlled studies designed to elucidate the impact of NRPT on endogenous estrogen levels and menopausal symptoms are clearly warranted.

## 5 Conclusion

In summary, evidence presented here demonstrates that supplementation with NRPT provides a safe, non-hormonal way of increasing endogenous E2/E1 ratio for women undergoing the menopause transition (perimenopause), mitigating undesirable menopausal symptoms. This work also expanded the number of NAD+ catabolites that can be analyzed in human urine.

## Data Availability

The raw data supporting the conclusions of this article will be made available by the authors, without undue reservation.
