# Supplementary material for: Nicotinamide riboside and pterostilbene reduces frequency and severity of undesirable symptoms of the menopause transition: an open-label, pilot clinical trial
Source: Front Aging. 2026 May 13;7:1773667. doi: 10.3389/fragi.2026.1773667 (PMC13213337; doi:10.3389/fragi.2026.1773667)
Supplement: Supplementary file 1 [file Presentation1.pptx]

## Slide 1
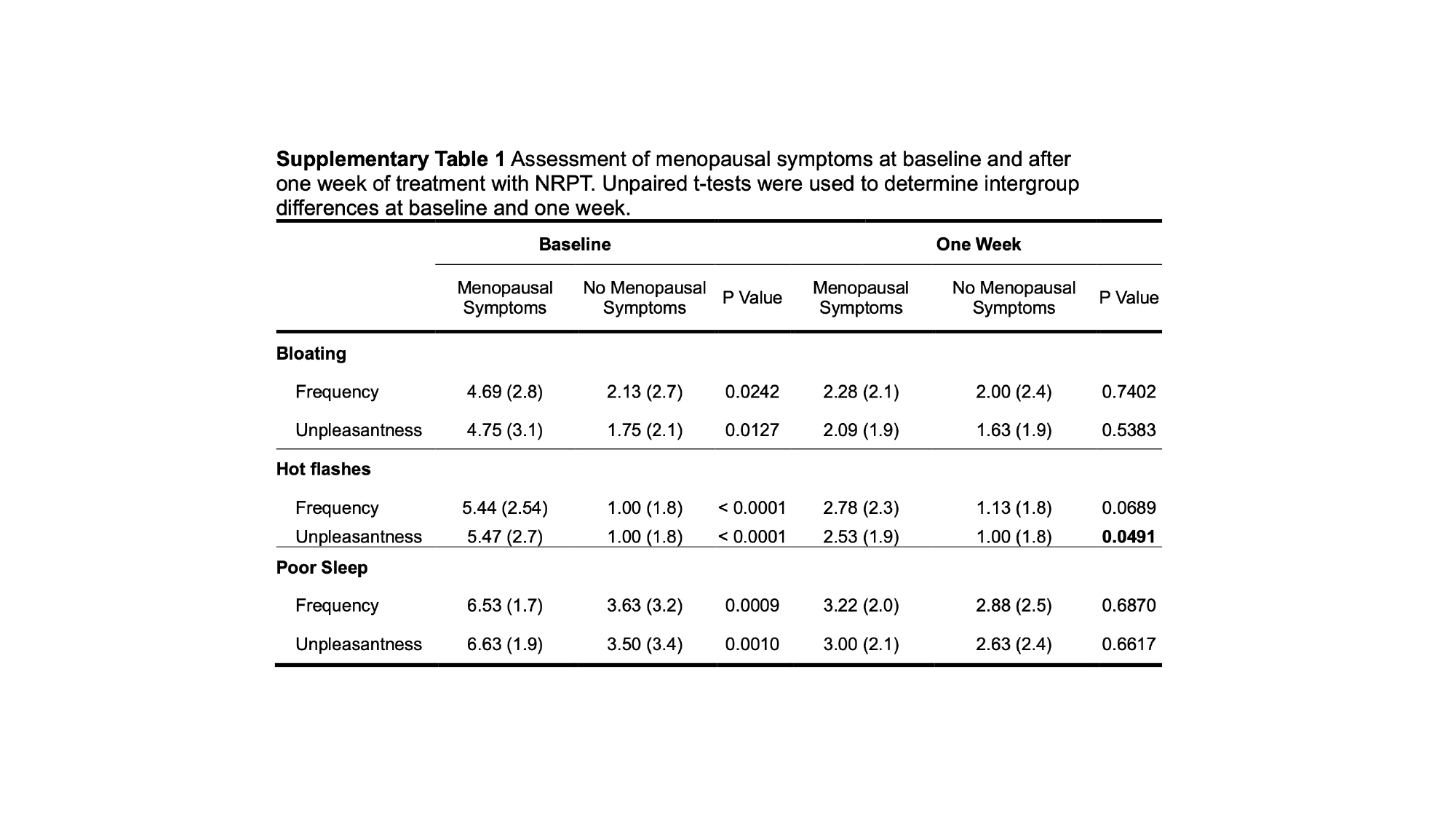

## Slide 2
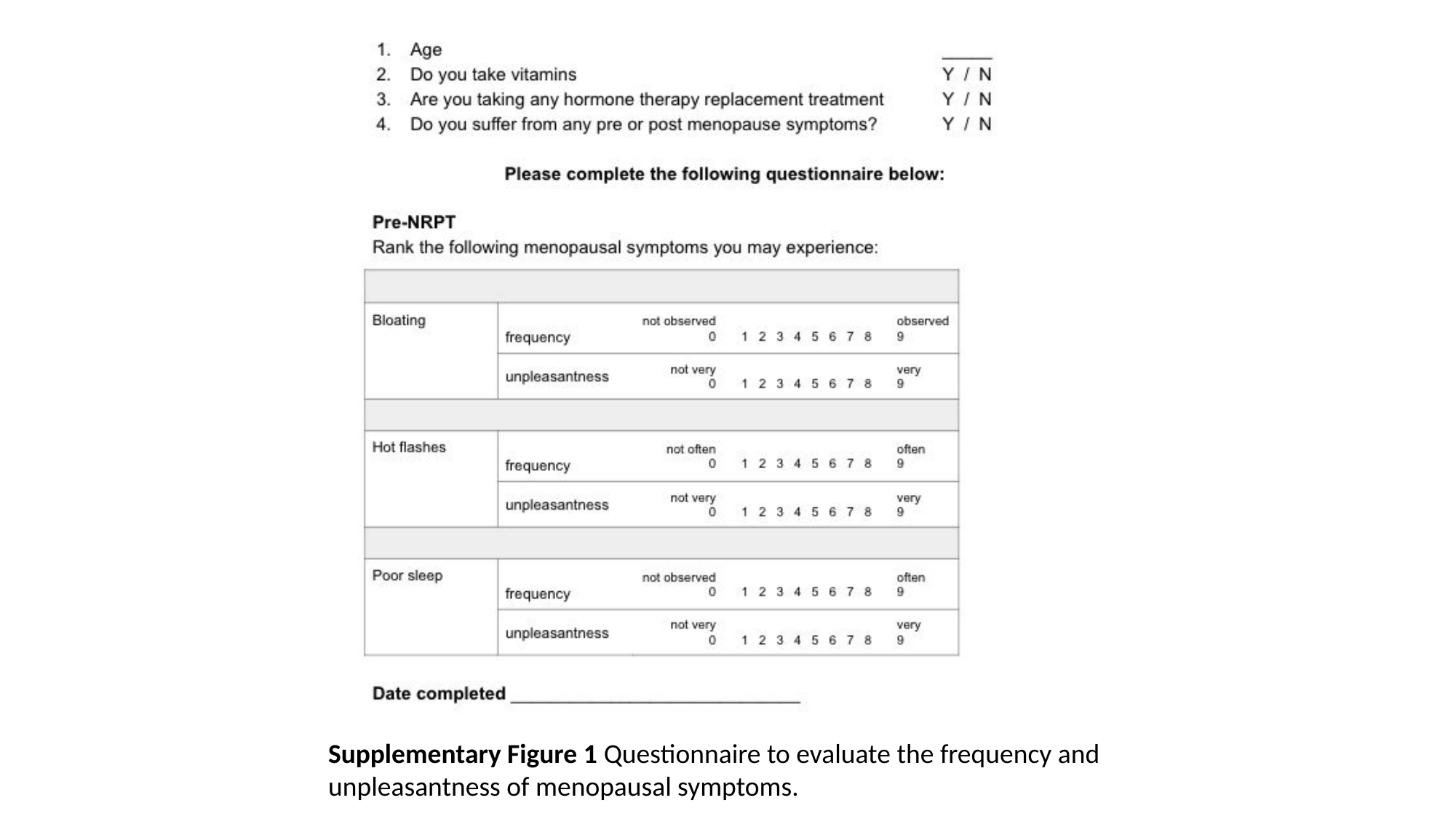

Supplementary Figure 1 Questionnaire to evaluate the frequency and unpleasantness of menopausal symptoms.

## Slide 3
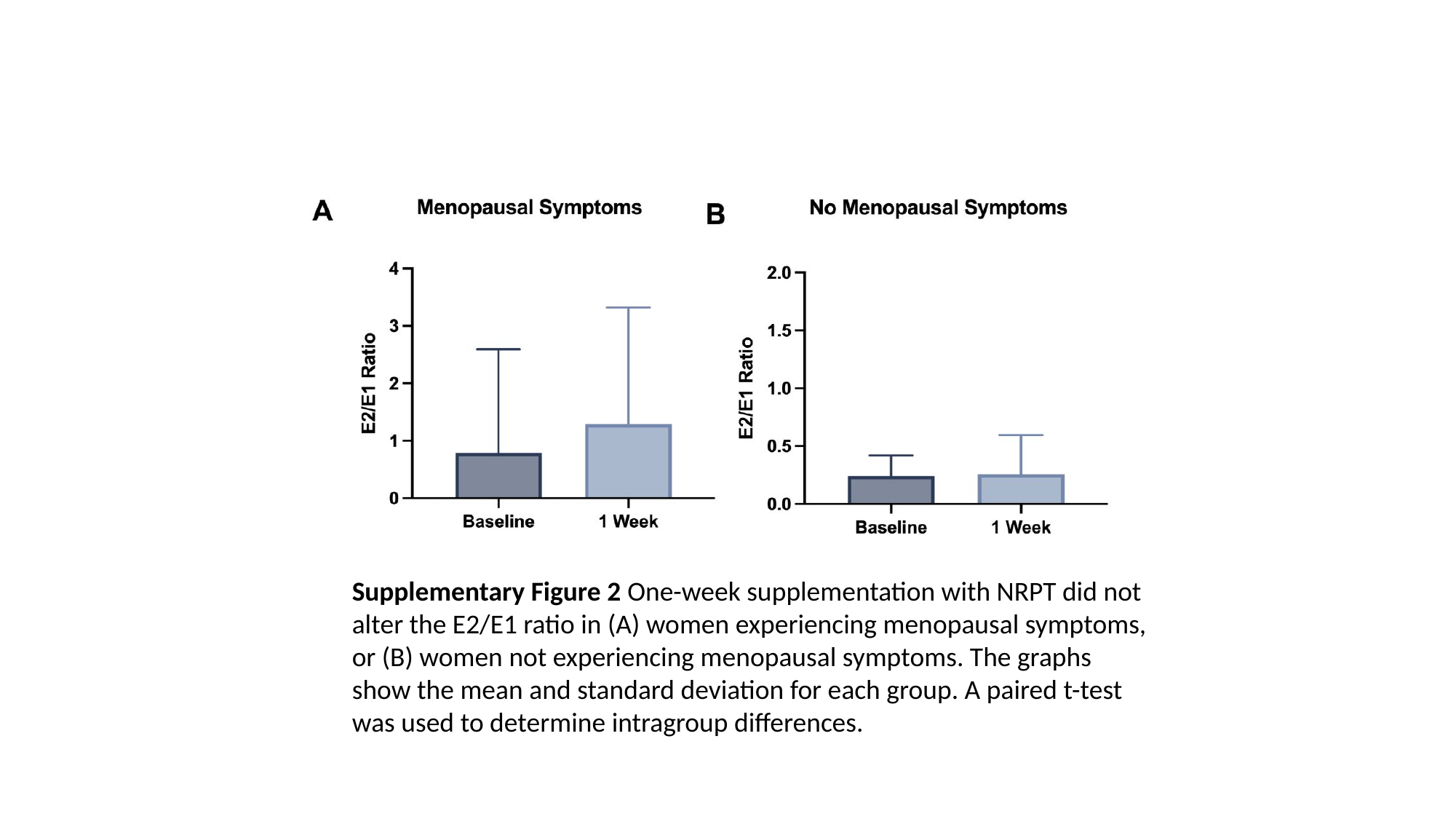

Supplementary Figure 2 One-week supplementation with NRPT did not alter the E2/E1 ratio in (A) women experiencing menopausal symptoms, or (B) women not experiencing menopausal symptoms. The graphs show the mean and standard deviation for each group. A paired t-test was used to determine intragroup differences.

## Slide 4
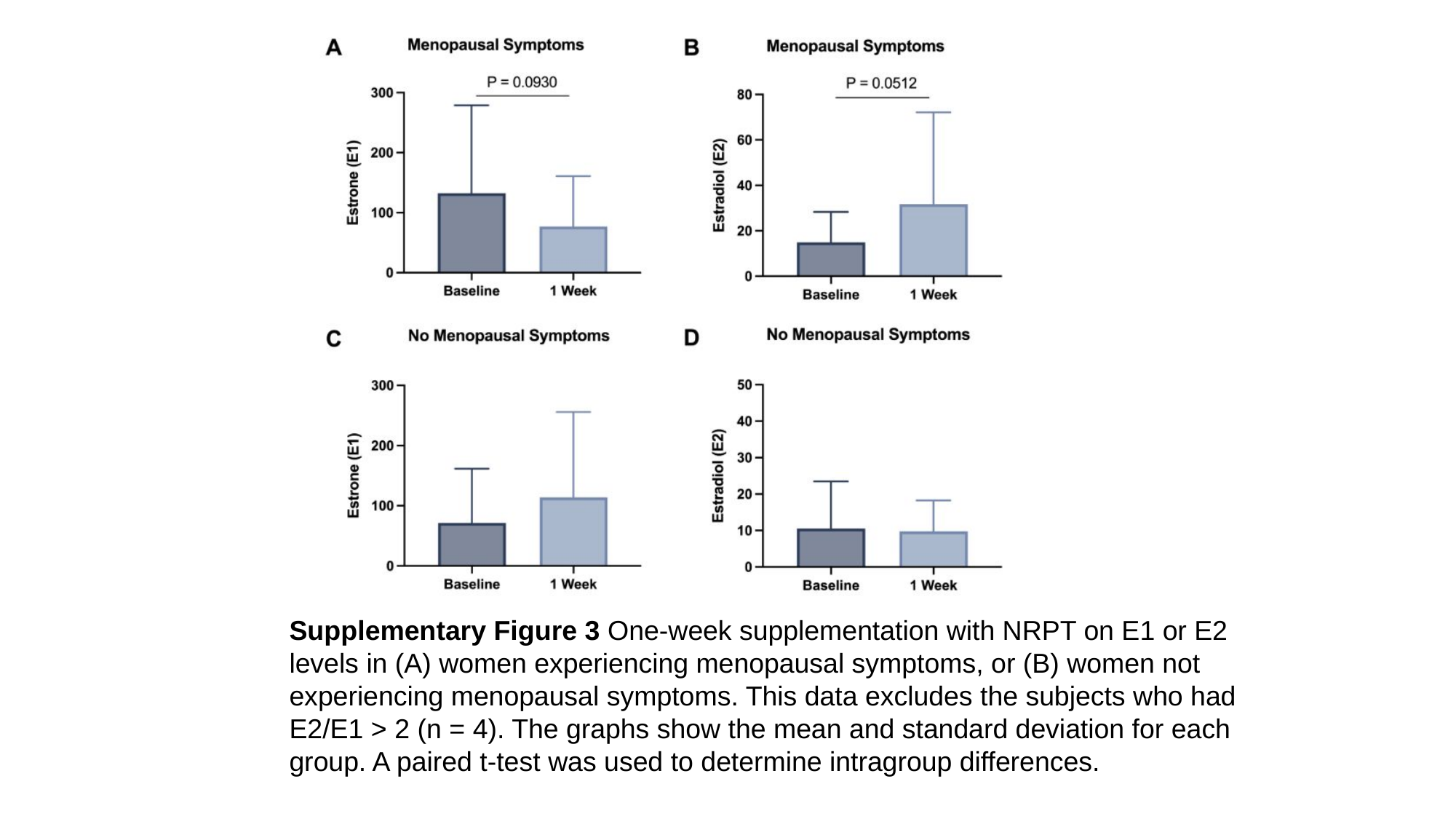

Supplementary Figure 3 One-week supplementation with NRPT on E1 or E2 levels in (A) women experiencing menopausal symptoms, or (B) women not experiencing menopausal symptoms. This data excludes the subjects who had E2/E1 > 2 (n = 4). The graphs show the mean and standard deviation for each group. A paired t-test was used to determine intragroup differences.

## Slide 5
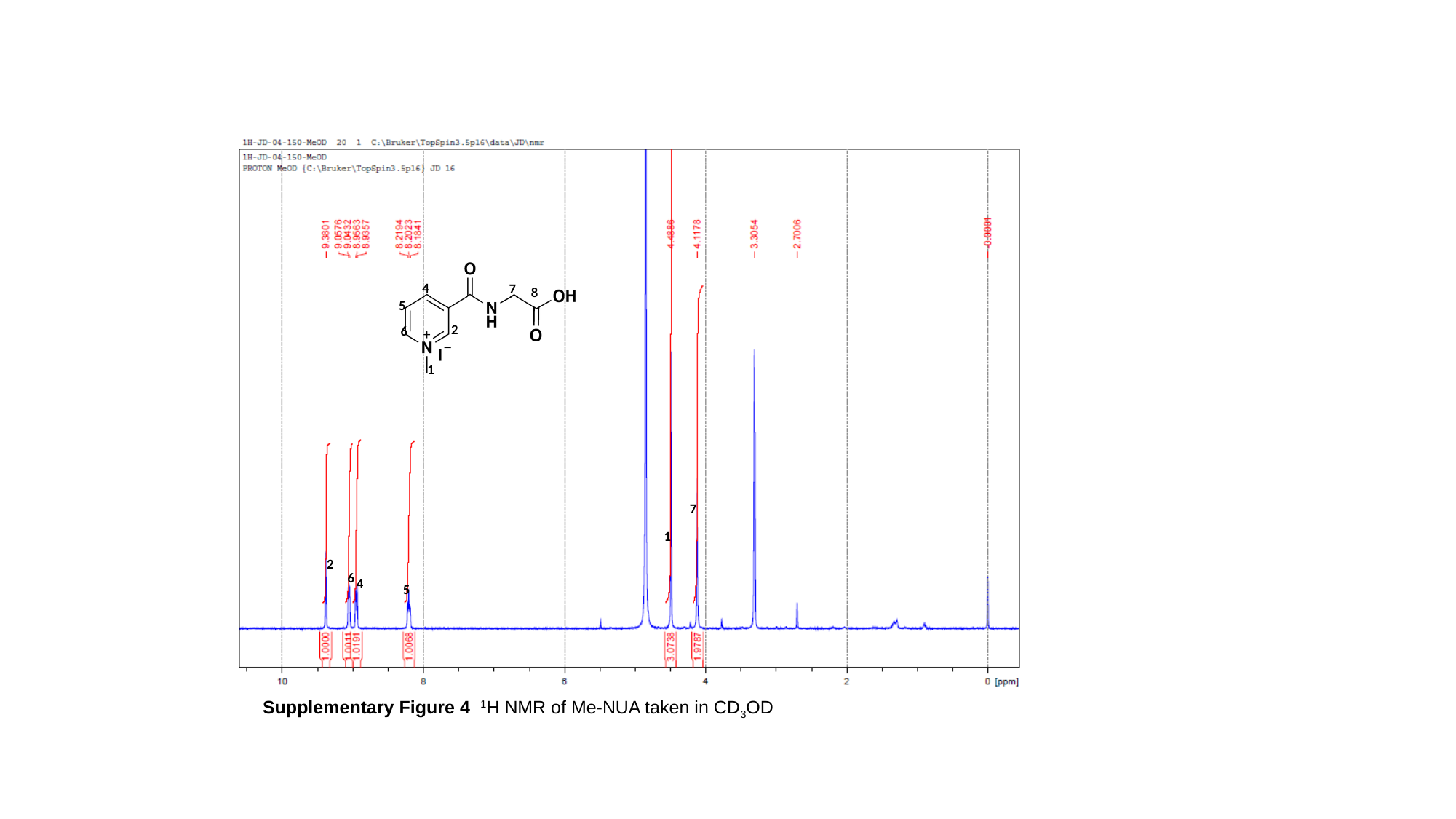

4
7
8
5
2
6
1
7
1
2
6
4
5
Supplementary Figure 4 1H NMR of Me-NUA taken in CD3OD

## Slide 6
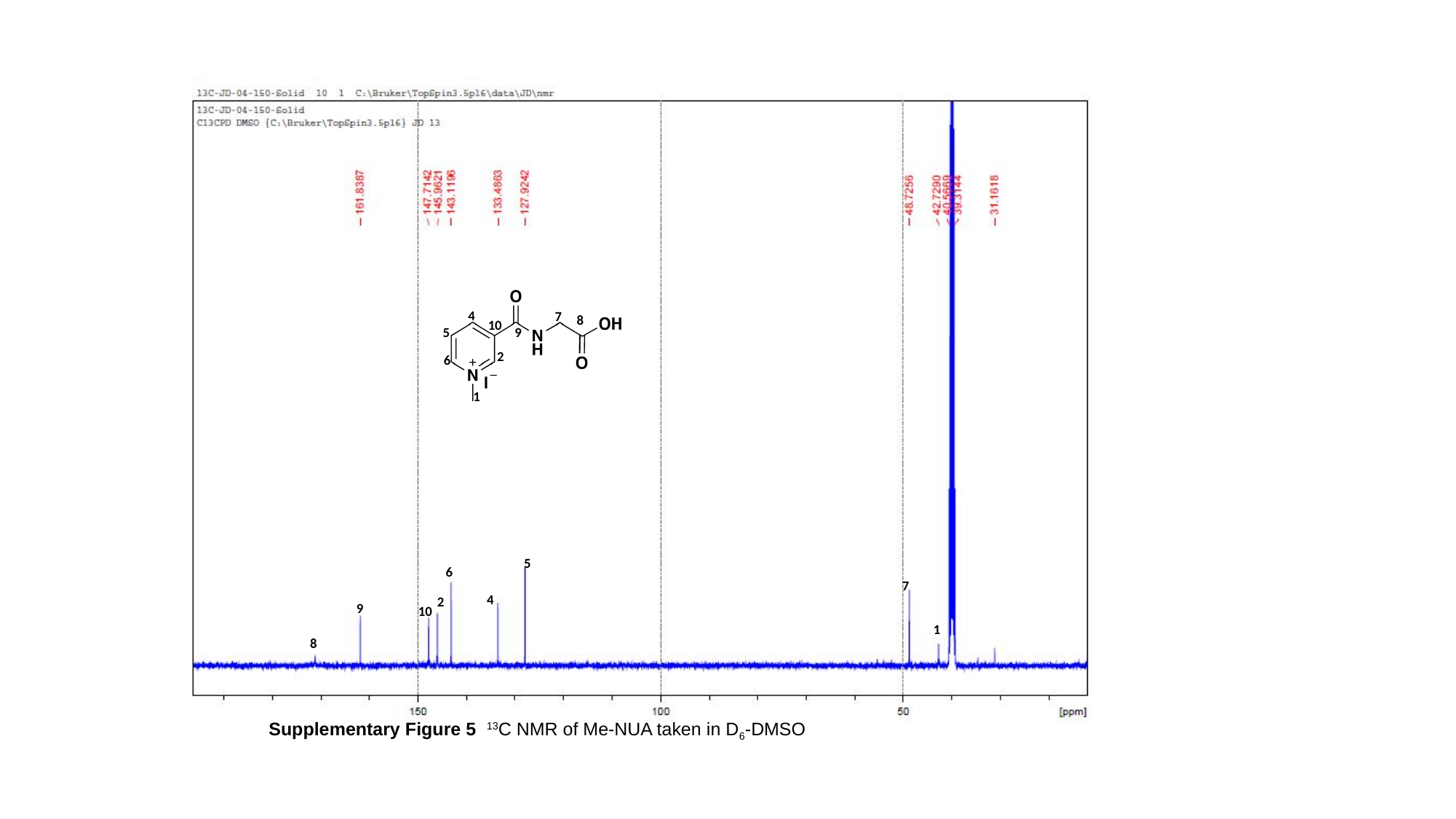

4
7
8
10
9
5
2
6
1
5
6
7
4
2
9
10
1
8
Supplementary Figure 5 13C NMR of Me-NUA taken in D6-DMSO

## Slide 7
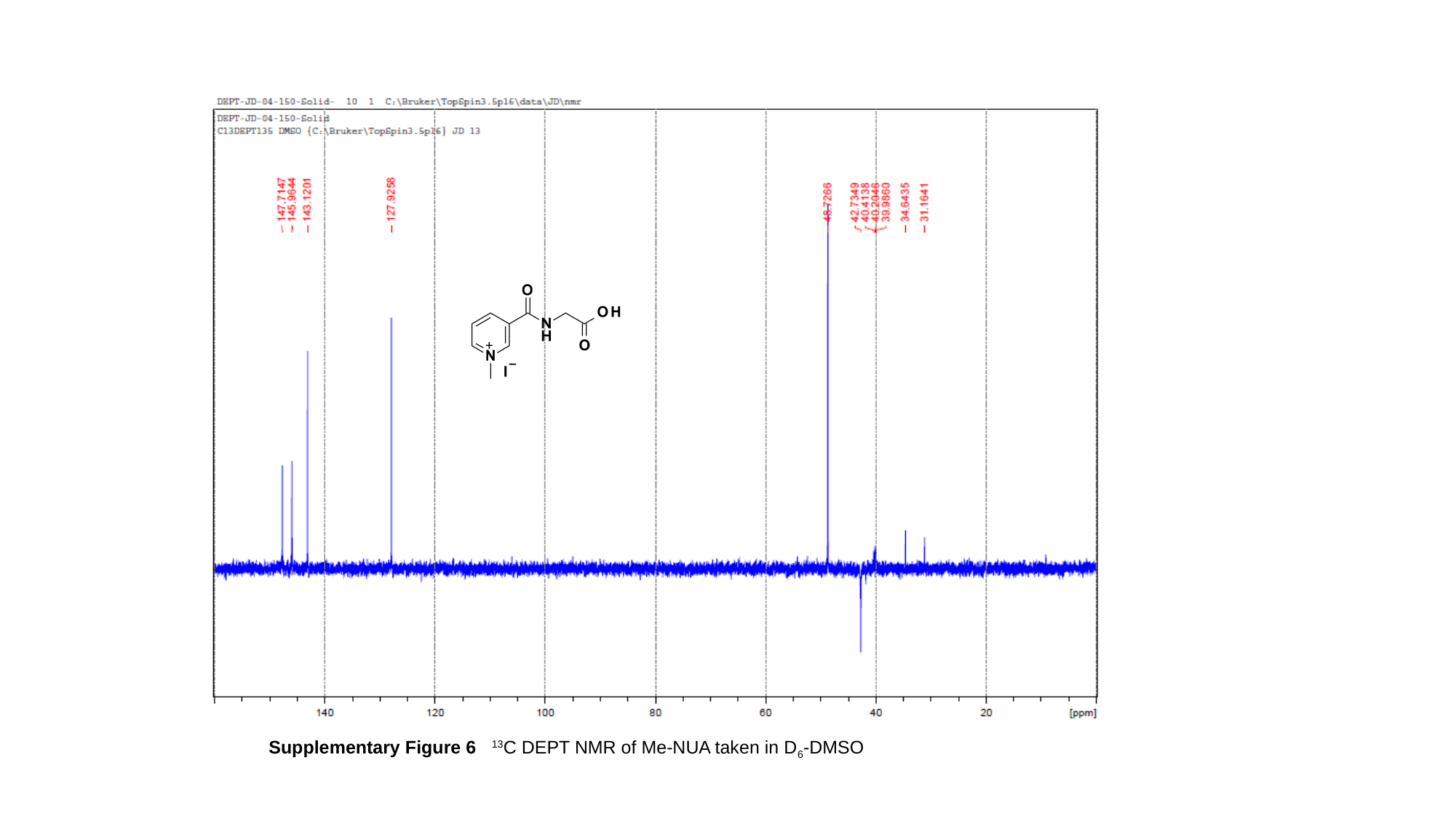

Supplementary Figure 6 13C DEPT NMR of Me-NUA taken in D6-DMSO

## Slide 8
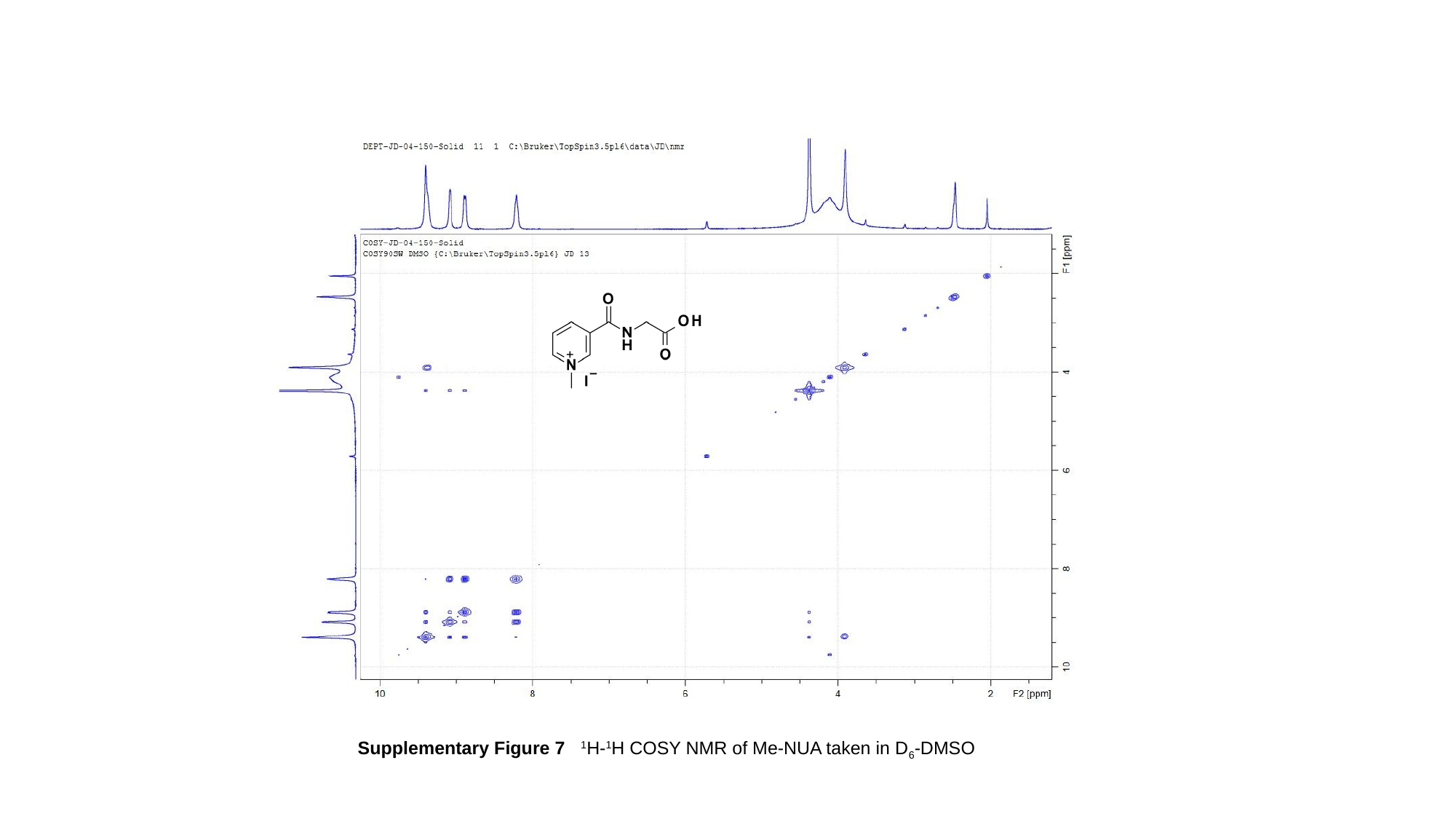

Supplementary Figure 7 1H-1H COSY NMR of Me-NUA taken in D6-DMSO

## Slide 9
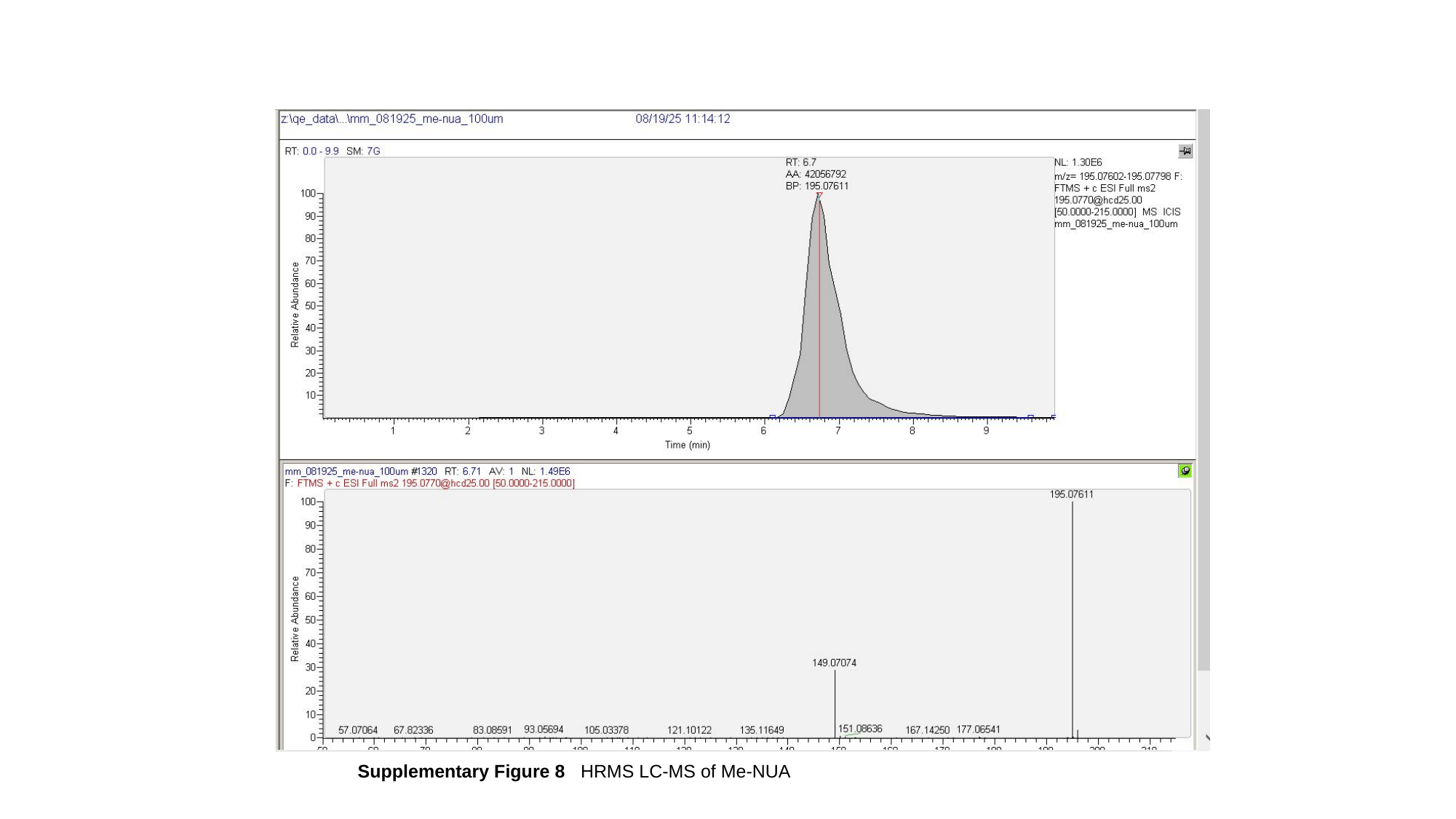

Supplementary Figure 8 HRMS LC-MS of Me-NUA
